# A Large-Scale Genome-Wide Association Analyses of Ethiopian Sorghum Landrace Collection Reveal Loci Associated With Important Traits

**DOI:** 10.3389/fpls.2019.00691

**Published:** 2019-05-29

**Authors:** Gezahegn Girma, Habte Nida, Amare Seyoum, Moges Mekonen, Amare Nega, Dagnachew Lule, Kebede Dessalegn, Alemnesh Bekele, Adane Gebreyohannes, Adedayo Adeyanju, Alemu Tirfessa, Getachew Ayana, Taye Taddese, Firew Mekbib, Ketema Belete, Tesfaye Tesso, Gebisa Ejeta, Tesfaye Mengiste

**Affiliations:** ^1^Department of Botany and Plant Pathology, Purdue University, West Lafayette, IN, United States; ^2^Malkassa Agricultural Research Center, Ethiopian Institute of Agricultural Research, Adama, Ethiopia; ^3^Chiro Agricultural Research Center, Ethiopian Institute of Agricultural Research, Chiro, Ethiopia; ^4^Bako Agricultural Research Center, Oromia Agricultural Research Institute, Bako, Ethiopia; ^5^School of Plant Sciences, Haramaya University, Dire Dawa, Ethiopia; ^6^Department of Agronomy, Purdue University, West Lafayette, IN, United States; ^7^Department of Agronomy, Kansas State University, Manhattan, KS, United States

**Keywords:** sorghum, population structure, genotyping-by-sequencing, genome-wide association study, compressed mixed linear model (CMLM)

## Abstract

The eastern Africa region, Ethiopia and its surroundings, is considered as the center of origin and diversity for sorghum, and has contributed to global sorghum genetic improvement. The germplasm from this region harbors enormous genetic variation for various traits but little is known regarding the genetic architecture of most traits. Here, 1425 Ethiopian landrace accessions were phenotyped under field conditions for presence or absence of awns, panicle compactness and shape, panicle exsertion, pericarp color, glume cover, plant height and smut resistance under diverse environmental conditions in Ethiopia. In addition, F1 hybrids obtained from a subset of 1341 accessions crossed to an A1 cytoplasmic male sterile line, ATx623, were scored for fertility/sterility reactions. Subsequently, genotyping-by-sequencing generated a total of 879,407 SNPs from which 72,190 robust SNP markers were selected after stringent quality control (QC). Pairwise distance-based hierarchical clustering identified 11 distinct groups. Of the genotypes assigned to either one of the 11 sub-populations, 65% had high ancestry membership coefficient with the likelihood of more than 0.60 and the remaining 35% represented highly admixed accessions. A genome-wide association study (GWAS) identified loci and SNPs associated with aforementioned traits. GWAS based on compressed mixed linear model (CMLM) identified SNPs with significant association (FDR ≤ 0.05) to the different traits studied. The percentage of total phenotypic variation explained with significant SNPs across traits ranged from 2 to 43%. Candidate genes showing significant association with different traits were identified. The sorghum *bHLH* transcription factor, *ABORTED MICROSPORES* was identified as a strong candidate gene conditioning male fertility. Notably, sorghum CLAVATA1 receptor like kinase, known for regulation of plant growth, and the *ETHYLENE RESPONSIVE TRANSCRIPTION FACTOR* gene RAP2-7, known to suppress transition to flowering, were significantly associated with plant height. In addition, the *YELLOW SEED1* like MYB transcription factor and *TANNIN1* showed strong association with pericarp color validating previous observations. Overall, the genetic architecture of natural variation representing the complex Ethiopian sorghum germplasm was established. The study contributes to the characterization of genes and alleles controlling agronomic traits, and will serve as a source of markers for molecular breeding.

## Introduction

Sorghum [*Sorghum bicolor* (L.) Moench] is the fifth most important cereal crop following wheat, rice, maize, and barley in both total production and acreage in the world (FAOSTAT, 2018). Sorghum is a staple food crop for millions of the most food-insecure poorest people in the semi-arid tropics of Africa, South Asia and Central America (Leff et al., 2004). These regions are often too dry for the cultivation of most of the other important cereal crops.

High genetic diversity is vital for the development of climate resilient crop varieties to mitigate the impact of climate change. As a major center of origin and diversity for sorghum (Dillon et al., 2007; Brenton et al., 2016), the extensive genetic variation of Ethiopian sorghums for traits including cold (Singh, 1985), and drought (Adugna, 2014) tolerance, resistance to grain mold and several foliar diseases (Weerasooriya et al., 2016) as well as nutritional quality (Rhodes et al., 2017) have been reported. Creating a better understanding of the genetic basis of all these traits is important for the improvement of this crop in the long-term. Traditionally, the identification of genomic regions and loci underlying traits of interest in crops were primarily based on evaluation of genetic populations derived from bi-parental crosses. However, this approach has yielded limited genomic resolution and restricted allelic diversity, as only allelic segregates between and among the parents of the particular recombinant progenies can be assayed (Korte and Farlow, 2013).

Genome-Wide Association Study (GWAS) is now more widely-used to identify candidate genes underlying traits of interest. With its power in overcoming the major limitations of bi-parental populations, it is becoming a more common approach in trait identification (Brachi et al., 2011) particularly with recent advances in high throughput DNA sequencing technologies, and large-scale precision-phenotyping. Genotyping-By-Sequencing (GBS) is a next-generation sequencing (NGS) based genotyping procedure that represent high-marker density approaches, and frequently used genotyping approach in GWAS. The GBS approach works by reducing genome complexity with restriction enzymes, combined with multiplex NGS for high-density single nucleotide polymorphism (SNP) marker discoveries (Elshire et al., 2011). The process associated with GBS including genome-wide molecular marker discovery, highly multiplexed genotyping, flexibility and low cost make it an excellent tool in studies of plant genetics and breeding (Deschamps et al., 2012; Poland and Rife, 2012). Understanding population genetic structure and familial relatedness among individuals of study materials are important procedure to undertake prior to GWAS analysis as these are sources of possible false-positives. Failures to account for population stratification and kinship diminish the revealing power of GWAS and can lead to spurious associations (Wu et al., 2011). It is therefore critical to choose appropriate models to reduce these two sources of false-positives.

Although the use of GWAS to delineate genomic regions with important traits in sorghum have been shown in several studies (Morris et al., 2013a; Adeyanju et al., 2015; Cuevas et al., 2017), huge gaps remain in our understanding of the genetic basis of many important traits in the crop. Previous GWAS studies in sorghum (Morris et al., 2013a,b; Cuevas et al., 2017) were mostly based on germplasm that have gone through the sorghum conversion program (Klein et al., 2008), an operation that converted tall, late, or photoperiod sensitive sorghums from the tropics into short, early, photoperiod insensitive lines. It has been adequately shown that the conversion process reduced genomic diversity in regions targeted for selection and hence limited success to dissect underlying loci for various traits in sorghum (Morris et al., 2013a). Herein, report findings from GWAS conducted on a large Ethiopian sorghum landrace collection with considerable genetic diversity that have evolved under the equally diverse environmental conditions in the region. In addition to describing the population structure and overall genomic diversity, several new loci and candidate genes underlying important agronomic, morphological, disease resistance and fecundity traits were identified. The candidate genes and genomic regions, once validated, can be utilized in marker assisted selection (MAS) to further enhance the efficiency of cultivar development.

## Materials and Methods

### Phenotyping

A total of 1425 Ethiopian sorghum landrace accessions were sampled from more than 9000 sorghum accessions maintained at the Ethiopian Biodiversity Institute (EBI) and the national agricultural research centers in Ethiopia. The selected accessions represent different sorghum growing regions and different agro-climatic zones. The global positioning system (GPS) was not captured at the time of collection but details on locality, agro-climatic information and race classification are included with the phenotype data that have been deposited on Purdue university repository^1^.

These materials were planted during 2015 and 2016 growing seasons at three different locations, Bako (9°08′N/37°03′E), Arsi Negelle (7°21′N/38°42′E) and Haramaya (9°24′N/42°01′E), representing three different climatic regions (Supplementary Table S1). Each accession was planted in a non-replicated single row of 3 m length with 20 cm distance between plants. Fertilizer was applied at the rate of 100 kg/ha DAP at planting and 50 kg/ha urea at knee height. Accessions were randomized in each test site.

Pure-lines in each accession were obtained by selecting and selfing five single heads representing the most frequent genotype within a row. The homogeneity was confirmed by planting the materials for at least one season. The five plants representing each accession were tagged and data on the following traits were collected following the standard descriptors used in sorghum germplasm characterization with some modifications (IBPGR and ICRISAT, 1993). Briefly, the data were scored as follows: (1) plant height in cm; (2) presence or absence of awns (1 = awned and 2 = awnless); (3) glume cover at maturity (1 = grain uncovered, 2 = 25% of grain covered, 3 = 50% of grain covered, 4 = 75% of grain covered, 5 = grain fully covered, 6 = glumes longer than grain); (4) pericarp color (1 = white, 2 = yellow, 3 = red, 4 = brown and 5 = buff); (5) panicle exsertion (1 = panicle well exserted with 10 cm between ligule of flag leaf to panicle base, 2 = 2–10 cm exsertion, 3 = less than 2 cm but ligule below the panicle base, 4 = peduncle recurved but panicle is below the ligule and clearly exposed splitting the leaf sheath, 5 = panicle covered by leaf sheath); (6) panicle compactness and shape (1 = loose erect, 2 = loose drop, 3 = compact elliptic (erect) and 4 = compact oval or recurved); (7) sorghum head smut (*Sporisorium sorghi*) damage score using a rating scale of 1–5, where 1 represents highly resistant and 5 is significant head damage; (8) male sterility. To study sterility reaction of the landraces, a total of 1341 accessions were initially crossed to an A1 cytoplasmic male sterile line, ATx623, during 2014 season and F1 seeds were harvested from each cross. All the F1 hybrids were grown on a single row plot during 2015 season, and three plants were carefully bagged before flowering. At flowering, individual plots were visually inspected for pollen shedding. At physiological maturity, the bags from all heads were opened and plants were carefully examined for seed set. Accessions whose hybrids completely failed to set seed from all bagged plants were designated as non-restorers (B) while those whose hybrids produced normal seed were designated as restorers (R). Others that have incomplete seed set were scored as partial restores (P). To reduce ambiguity, accessions with clear-cut fertility or sterility responses scored as either B or R were considered for GWAS analysis.

### DNA Extraction and Genotyping by Sequencing

DNA was extracted from a week-old freeze dried seedlings representing one of the five tagged plants of 1425 accessions following acetyl trimethylammonium bromide (CTAB) protocol modified for 96-well plates (Mace et al., 2003). A total of fifteen 96-plex GBS libraries were constructed and genotyped at the University of Wisconsin, Biotechnology center. The genotyping by sequencing (GBS) procedure (Elshire et al., 2011) was implemented using the ApeKI restriction enzyme (recognition site, G| CWCG). The GBS library was sequenced on Illumina HiSeq 2500 lane following the manufacturer’s protocol. The SNP datasets generated for this study can be found in Purdue university repository^2^.

### SNP Calling and Quality Control

Single nucleotide polymorphism (SNP) calling was performed with TASSEL GBS pipeline v5 (Glaubitz et al., 2014) based on alignment to the reference genome of *Sorghum bicolor* version 3.1.1 (McCormick et al., 2018) accessible on Phytozome (Goodstein et al., 2012). Resulting SNPs were further processed using PLINK version 1.90 (Purcell et al., 2007) by removing those with MAF of less than 0.05, individuals with less than 20% and loci with less than 40% missing.

### Data Analysis

#### Phenotypic Data Analysis

The phenotypic data collected included ordinal categorical (panicle exsertion and glume cover), nominal categorical (pericarp color and panicle compactness and shape), binary (presence or absence of awns and fertility reactions) and quantitative trait (plant height and smut damage score). The frequency distribution of the categorical data across environments were visualized using histogram. Analysis of variance was conducted for plant height and smut damage score to assess the proportion of genotype to the total phenotypic variation and residual error. To partition the different variance components attributing to phenotypic variation, we implemented linear mixed effects model using an R software (R Core Team, 2017) package called lmer4 (De Boeck and Wilson, 2004). Heritability for quantitative traits was also determined by dividing the variance due to genotype by the total variance.

#### Cluster Analysis and PCA

To describe population structure in the Ethiopian sorghum landrace collection, pairwise distance-based hierarchical clustering was conducted by calculating genetic distance (identity-by-state, IBS) based on 72,190 SNP markers in PLINK version 1.90 (Purcell et al., 2007). A Ward’s minimum variance hierarchical cluster dendrogram was then built from the IBS matrix using the R (R Core Team, 2017) package, Analyses of Phylogenetics and Evolution (APE) (Paradis et al., 2004). Secondly, model-based maximum likelihood estimation of individual ancestries from multi-locus SNP genotype datasets using ADMIXTURE 1.3.0 (Alexander and Lange, 2011) was used to identify ancestries of each sorghum accession. The Admixture analysis was performed for different K (number of sub-populations) varying from 2 to 20. The most appropriate *K*-value was selected after considering (i) 10-fold cross-validations whereby the best K exhibits low cross-validation error compared to other *K*-values and (ii) good correspondence with the clustering pattern obtained by hierarchical cluster tree.

To further understand the pattern of genetic relatedness across accessions, principal components analysis (PCA) was conducted using the ggplot2 package (Wickham, 2009) in R software (R Core Team, 2017). PCA was considered as an effective approach to diagnose population structure; and the first two axes of the PCA were used to draw a scatter plot to visualize genetic differentiation among genotypes.

#### Genome-Wide Association Analysis (GWAS)

Phenotype data from 1425 sorghum accessions, for all the traits except sterility reaction, and a total of 72,190 robust SNP markers generated from GBS data were used for GWAS analysis. For sterility reaction a total of 1002 accessions categorized as fertile or sterile and fulfilled SNP quality parameters were used. In GWAS, the presence of population structure and kinship often lead to spurious associations (Wu et al., 2012), and should be adequately accounted for in choosing a GWAS model. Hence, an association analysis was performed with a compressed mixed linear model (CMLM) (Zhang et al., 2010) implemented in the GAPIT package (Lipka et al., 2012) in R software (R Core Team, 2017) using 72,190 SNPs with a MAF ≥ 0.05. Kinship was calculated as per procedure described by VanRaden (2008), and a co-ancestry matrix from ADMIXTURE was included as a covariate in GAPIT to reduce spurious associations. Log Q–Q plots of *p*-values were examined to determine how well the models accounted for population structure and familial relatedness. GWAS was performed both as cumulative and separately for each location/year across all the traits. Significant associations were determined for each trait using a false discovery rate- adjusted *p* < 0.05 as implemented in GAPIT. The Bonferroni error rate control known to extremely increase false negatives were also included in the analysis for comparison. Manhattan and Q–Q plots were visualized using the R package qqman (Turner, 2014). All the significant SNP markers were mapped onto *Sorghum bicolor* v3.1.1 (McCormick et al., 2018) genome-based on the physical position obtained during SNP calling for each of the SNPs in Phytozome v12.1 (Goodstein et al., 2012) using JBrowse (Skinner et al., 2009).

#### Linkage Disequilibrium (LD)

LD heat map package (Shin et al., 2006) in R software (R Core Team, 2017) were used to perform LD and produced a graphical display, as a heat map, of pairwise LD measurements among SNPs with significant association for each of the traits independently.

## Results

### Phenotypic Variation

The Ethiopian sorghum landrace showed high variability in plant height (range = 90–533 cm, mean = 323.31 cm) among accessions (Supplementary Figure S1). Analysis of variance revealed that phenotypic variation due to environment as well as G × E interaction were lower for plant height, with genotypic differences being the primary sources of variation. A reproducibility value of 0.76 for plant height also indicated that observations across different environments were consistent (Table 1). In contrast, more than 65% of the variance associated with smut disease incidence was attributed to differences in environment and G × E interaction effects, indicating large variations in disease severity across environments (years) for this trait. Reproducibility for smut resistance showed a low value of 0.34 (Table 1). The low reproducibility in the smut disease response of genotypes across environments could be attributed to the greater effect of the environment on the incidence and severity of the disease. All phenotypic data, both categorical and quantitative traits, analyzed in the study were summarized and presented in histograms (Supplementary Figure S1).

**Table 1:** Estimation of variance components using linear mixed effects model (lme) for plant height and smut damage score in 1425 Ethiopian sorghum landrace accessions across different environments.

Traits	Variance components	Variance estimate (δ^2^)	Standard Deviation	Total contribution to variance in percent	Heritability
Plant height	Genotype	3543	59.52	75.79	0.76
	Residual	1132	33.64	24.21	
	Total	4675		100.00	
Smut score	Genotype	0.15	0.01	34.02	0.34
	Residual	0.28	0.02	61.98	
	Total	0.43		100	


### SNP Markers and Allele Frequency

**FIGURE 1:**
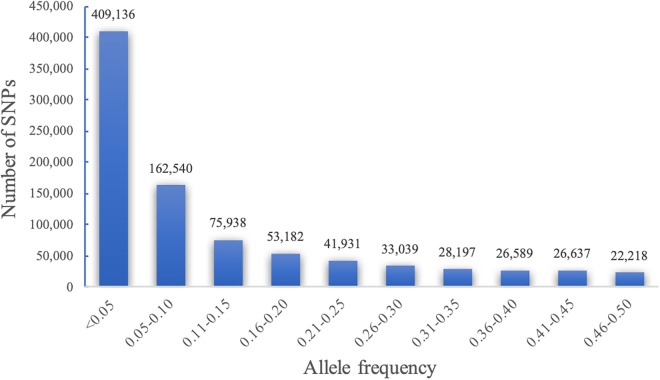
Minor allele frequency (MAF) and number of SNPs based on 1425 unique sorghum accessions from Ethiopia.

Quality control (QC) of genotypic data is a key step in GWAS. It involves removing SNPs with high rate of missing genotype/markers and setting a minimum minor allele frequency (MAF). Initially, 879,407 SNP markers were discovered from 1428 accessions genotyped. The quality control of SNP data based on individuals with <20% and markers with <40% missing and MAF greater than 0.05 produced 72,190 robust SNP markers with 1425 accessions passing the criteria at 95% genotyping rate. Allele frequency is also important in GWAS because genetic associations with SNPs having very low MAF can give false positive results. Our analyses revealed that 46% of the SNPs called in our study represented rare alleles with MAF < 0.05 (Figure 1).

### Population Structure

**FIGURE 2:**
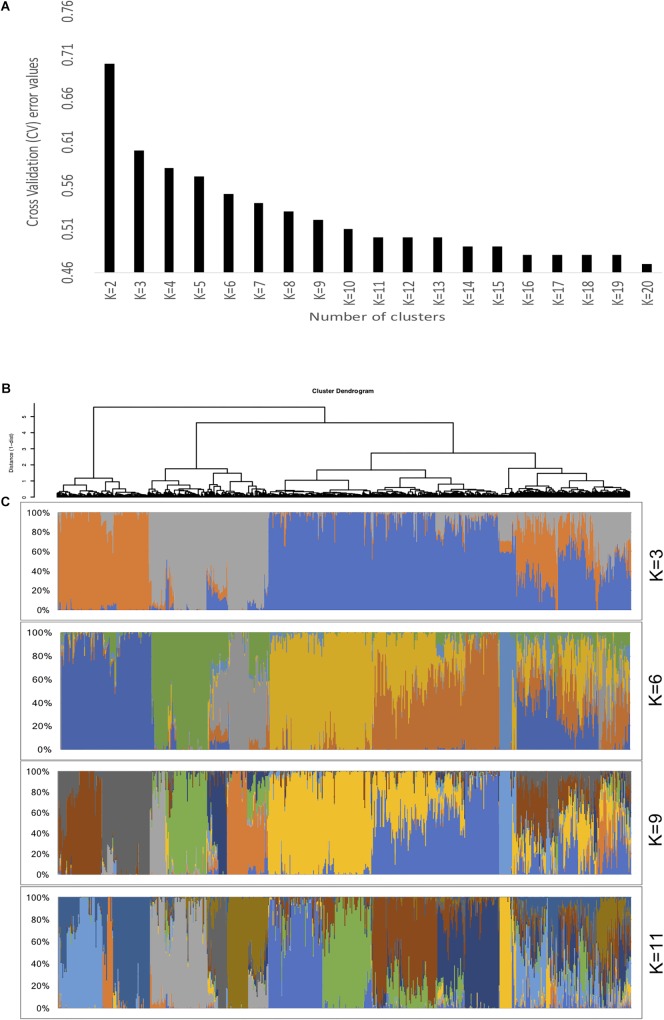
Population genetic structure of the Ethiopian sorghum landrace collection. **(A)** A 10-fold cross-validation (CV) error rates for *K* = 2 to *K* = 20 determined by ADMIXTURE analysis. The CV values of different K indicated steep decrease until it reached 11, showing optimal number of clusters as *K* = 11, **(B)** Hierarchical clustering (Ward’s minimum variance method) dendrogram, **(C)** Individual ancestry estimated based on ADMIXTURE analysis for different *K*-values. Each individual accession is represented as a single vertical line partitioned into segments corresponding to the inferred membership according to different K genetic clusters as indicated by the colors.

**FIGURE 3:**
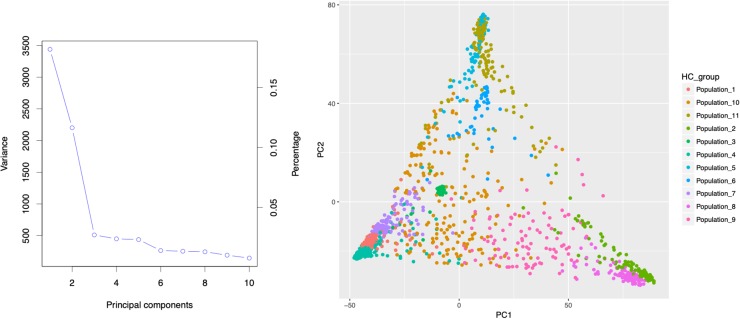
Principal component analysis of 1425 Ethiopia sorghum landrace accessions based on 72,190 high quality SNPs with MAF > 0.05 using the first two principal components. The large proportion of the of the variances contained in the data are retained by the first two principal components as indicated on the scree plot.

Population stratification results in differences in allele frequencies between subpopulations due to ancestral differences. When not accounted properly in GWAS analysis, it can cause linkage disequilibrium (LD) between unlinked loci consequently generating spurious marker-trait associations. In the current study, the ADMIXTURE analysis using 10-fold cross validation (CV) for *K* = 2 to *K* = 20 indicated steep decrease until *K* = 11, showing optimal number of sub-population at *K* = 11 (Figure 2). About 65% of the genotypes (926 accessions) assigned to either one of the 11 sub-populations (clusters) had high ancestry membership coefficient with the likelihood of more than 0.60. The remaining 35% represented accessions with high admixture. The principal component analysis (PCA) using two PCs further described the population stratification in the collection. A scree plot generated to visualize the fraction of variance represented by each of the 10 principal components, showed that two of the principal components (PC1 and PC2) explained the highest proportion of the total variance (Figure 3).

### GWAS for Important Traits in Sorghum

**FIGURE 4:**
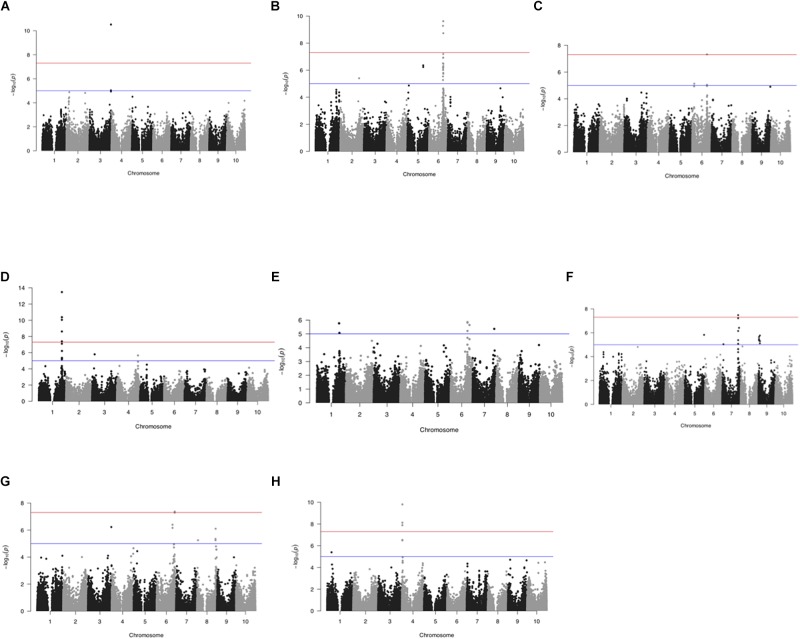
GWAS across 1425 Ethiopian sorghum landrace collection using 72,190 SNP markers. Manhattan plots showing significant false discovery rate (FDR)- adjusted *P*-value of ≤0.05 associated with different traits using CMLM. **(A)** awns, **(B)** panicle compactness and shape, **(C)** panicle exsertion, **(D)** pericarp color, **(E)** glume cover, **(F)** plant height, **(G)** resistance to smut, and **(H)** male sterility. The horizontal red and blue lines represent FDR adjusted *p*< 0.01 and < 0.05, respectively.

GWAS identified 102 different SNPs with significant association (FDR ≤ 0.05) to plant height, presence or absence of awns, glume cover, pericarp color, panicle compactness and shape, panicle exsertion, smut resistance and male sterility (Figure 4 and Supplementary Figure S2). At the same time, the Bonferroni error rate control reduced the total SNPs to only 47 (Supplementary Table S2). This error rate control method is usually known for leading to high rate of false negatives. Presumably, some important genes such as TANNIN 1 previously reported to have association with pericarp color (Wu et al., 2012) were not significant with Bonferroni. Hence, we considered FDR in further analysis to reduce possible exclusion of some important SNPs. Based on the available sorghum reference genome sequence data, candidate genes containing most likely SNPs for each trait were identified. The SNPs represented 61 candidate genomic regions associated with the studied traits revealing the genetic architecture across the genome (Table 2).

**Table 2:** Summary of significant single nucleotide polymorphisms (SNPs) representing different regions across sorghum chromosome for the eight morphological, agronomic, smut resistance, and male sterility.

Traits	Chromosome	Number of chromosomal regions	False discovery date (FDR)	*P*-value	*R*^2^
Presence or absence of awns	3	1	*q* ≤ 0.00000028	*p* ≤ 3.05000E-11	0.2404
Panicle compactness and shape	2,5,6	15	*q* ≤ 1.47650E-05	*p* ≤ 4.38953E-10	0.3432–0.3530
Panicle exsertion	6	2	*q* ≤ 0.00345773	*p* ≤ 4.78977E-08	0.2371
Pericarp color	1,3,4	8	*q* ≤ 2.4055E-09	*p* ≤ 3.33216E-14	0.1096–0.1345
Glume cover	6	4	*q* ≤ 0.03128427	*p* ≤ 1.38672E-06	0.1804–0.1809
Plant height	2,5,7,8,9	12	*q* ≤ 0.00218362	*p* ≤ 3.33852E-08	0.4127–0.4173
Smut resistance score	3,6,8	10	*q* ≤ 0.0007417	*p* ≤ 4.27118E-08	0.0162–0.0239
Sterility reaction	1,4	9	*q* ≤ 0.0000114	*p* ≤ 1.58000E-10	0.2080–0.2358


### Awns

**FIGURE 5:**
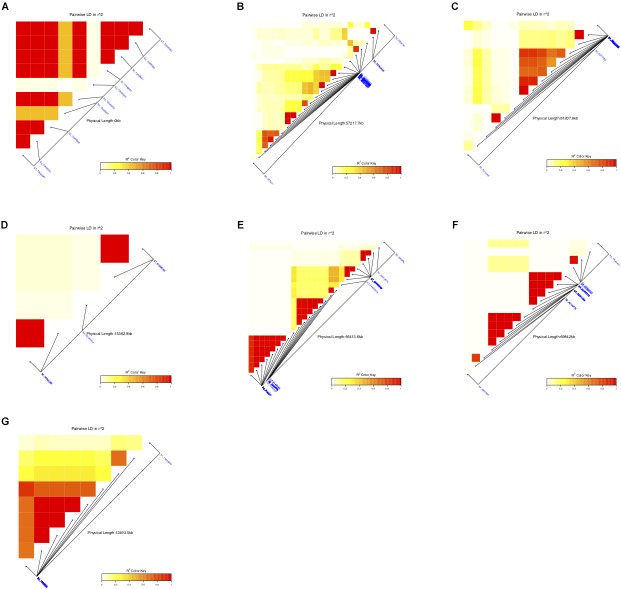
Summary of the local LD and haplotype blocks for different traits **(A)** awns, **(B)** panicle compactness and shape, **(C)** pericarp color, **(D)** glume cover, **(E)** plant height, **(F)** resistance to smut, and **(G)** male sterility. The R^2^ color key indicates the degree of significant association.

The association study based on absence or presence data for awns identified eight SNPs with significant association to this trait (FDR < 0.01) at 72.6 Mb on chromosome 3. These SNPs are also highly linked, with strong LD (Figure 5) and explained 24.04% of the total phenotypic variation. The SNPs were all located within the gene model Sobic.003G421300 on chromosome 3 annotated to encode a protein of unknown function (DUF640) (DUF640, which is homologous to the rice gene Oropetium_20150105_15608 (Similar to G1L2: Protein G1-like2 (Oryza sativa subsp. japonica).

### Panicle Compactness and Shape

A total of 24 SNPs on chromosome 2, 5, and 6 were significantly (FDR ≤ 0.05) associated with panicle compactness and shape. These SNPs accounted for up to 35.30% of the total phenotypic variance for the trait. In total, 15 regions across the genome were identified as having significant association with panicle compactness and shape. Among these, three genes Sobic.006G093400, Sobic.006G097900, and Sobic.006G096500 encode putative uncharacterized proteins and six encode proteins that share similarity to characterized proteins. Sobic.006G087900 encodes a putative *Serine/threonine-protein kinase*-like (CCR4*)* while *Sobic.006G091000* encodes a putative PHOSPHATASE METHYLESTERASE 1. A putative signal recognition particle 19 kDa protein encoded by Sobic.006G099800, previously linked to thermo tolerance was also identified. In addition, other genes in close-proximity to the most significant SNPs were identified including a *ternary complex factor, MADS* box interacting protein and a putative protein with 90% homology to *Plastocyanin*-like domain.

### Panicle Exsertion

A single SNP (S6_46493884) within Sobic.006G094800 encoding a protein with no predicted or functionally defined domain, was identified on chromosome 6 for significant association with panicle exsertion. The SNP explains 23.71% of the phenotypic variation for panicle exsertion. Another gene model, Sobic.006G094600, encoding putative *LSM1* (Sm like proteins) protein was also identified adjacent to Sobic.006G094800.

### Pericarp Color

GWAS for pericarp color identified a total of 15 SNPs across three chromosomes (1,3 and 4) significantly associated with pericarp color, most of them being located within genes of predicted functions. of these 13 SNPs implicated in pericarp color are from chromosome 1 and displayed strong LD showing high linkage (Figure 5), whereas, the remaining two SNPs are from chromosome 3 and 4. The R^2^ explaining the total phenotypic variation of the trait ranges between 10.96 and 13.45%. Five of the SNPs are in the candidate gene similar to *YELLOW SEED1 (*Sobic.001G397900). This locus contains three genes encoding the MYB transcription factor *YELLOW SEED1* (*Y1*, Sobic.001G398100), similar to YELLOW SEED1 and a third highly related MYB, likely to be a pseudogene. In addition, four additional loci harboring the gene models (Sobic.001G398100, Sobic.001G400400, Sobic.001G400300, and Sobic.001G397850), two novel loci on chromosome 3 and chromosome 4 were significantly associated with pericarp color. The Sobic.004G280800 gene encodes a protein with high sequence identity (94.3%) to the maize WD repeat containing protein encoded by *TRANSPARENT TESTA GLABRA* *1 (TTG1)* gene. In addition, Sobic.001G400500, adjacent to Sobic.001G400400, predicted to encode a pectinestrase is another candidate gene associated with pericarp color.

### Glume Cover

A total of three loci including two at 45.6 and 52.18 Mb on chromosome 6 and one at 61.9 Mb on chromosome 1were significantly associated with glume cover. The R^2^ explaining the total variance in glume cover for all the SNPs is about 18%. SNPs within Sobic.006G095550, with no functional annotation, and a *PEPTIDYL-PROLYL CIS-TRANS ISOMERASE* (Sobic.006G095400) gene, AN *INDOLE-3-ACETIC ACID-AMIDO SYNTHETASE GH3.8 (Sobic.001G331200*) all show significant association with glume cover. In addition, adjacent to Sobic.006G095550, candidate genes *FKBP77* (Fruktokinase binding proteins 77) and a *PENTATRICOPEPTIDE REPEAT (PPR)* gene were associated with glume cover.

### Plant Height

A total of 26 SNPs with significant association to plant height were identified with more than half (14 SNPs) on chromosome 9 followed by eight SNPs on chromosome 7, two on chromosome 8, single SNPs each on chromosome 5 and chromosome 2. The R^2^ indicating percent total phenotypic variance explained across the most significant SNPs is about 41%. The study also identified several new loci in 12 regions to be associated with plant height on chromosome 2, 5, 7, 8, and 9. The candidate genes defined by the SNPs associated with plant height encode a MYB domain protein *110*, *LIPOATE-PROTEIN LIGASE*, and *CHLOROPHYLL A/B-BINDING* protein precursor. Two genes from chromosome 9, associated with plant height, were *DIHYDROLIPOYL DEHYDROGENASE (Sobic.009G052200)* and *ETHYLENE-RESPONSIVE TRANSCRIPTION FACTOR (RAP2-7, Sobic.009G024600)*. Furthermore, *CLAVATA1* receptor like kinase (*Sobic.002G172100*), T*YROSINE/NICOTIANAMINE AMINOTRANSFERASE* (*Sobic.005G200300*) and C*YTOCHROME P450 (Sobic.008G058500*) were identified. Adjacent to the most significant SNPs, two candidate genes, *Sobic.007G161800* and *Sobic.008G058600* encoding Phosphofructokinase (PfkB) type carbohydrate kinase protein and a universal stress protein family from chromosome 7 and 8 respectively were identified.

### Smut Resistance

A total of 17 SNPs with significant association (FDR ≤ 0.05) to smut resistance were discovered based on our GWAS analysis. Of which, 12 SNPs were from chromosome 6, four on chromosome 8 and one on chromosome 3. The majority of the SNPs are associated with genes that have predicted functions (Supplementary Table S2). The *R*^2^-value of all the SNPs reported here are the lowest among the studied traits, explaining only about 2% of the total phenotypic variance for smut resistance. Several candidate genes including GLYCOSYLTRANSFERASE 3 and *MYB* transcription factor *(Sobic.008G035800)*, *MADS box protein (Sobic.006G189500)*, autophagy-related protein 8C precursor (*Sobic.006G220900*), and *RWP-RK* domain *(Sobic.006G133900)* were identified. RWP-RK proteins have a key role in regulating responses to nitrogen availability (Guo and Schnurbusch, 2016). In addition, based on significant SNPs, the candidate genes *AMINOMETHYLTRANSFERASE* (*Sobic.006G220800*), lipoate-protein ligase B containing protein (*Sobic.008G15200*) and a *TRANSCRIPTION TERMINATION FACTOR 2* (*Sobic.003G428100*) were identified.

### Restoration of Male Sterility

GWAS on the male sterility trait showed significant association in two major regions on chromosome 1 and 4. The different loci associated to male sterility were represented by nine SNPs, eight on chromosome 4 and one single SNP on chromosome 1. Most SNPs associated with male sterility fell in regions with predicted functions but two represented uncharacterized proteins. The *CALMODULIN-LYSINE N-METHYLTRANSFERASE* (*Sobic.004G017100*) with significant identity to the rice Os09g0543100, previously implicated in abiotic stress responses was identified. The eight SNPs associated with male sterility on chromosome 4 spanned a 44.5 kb genomic region with high LD (Figure 5), indicating tighter linkage. R^2^ explaining the total variation in male sterility with these SNPs ranged from 20.80 – 23.58%. The strongest SNP (S4_1379552) was located near Sobic.004G017500, a candidate gene which encodes a putative *BHLH* transcription factor with high homology to *ABORTED MICROSPORES (AMS)*. We have also identified Sobic.001G166200, annotated as a zinc finger family protein with candidate genes (Sobic.001G166300, Sobic.001G166401, and Sobic.001G166500) with 96% sequence homology to *oxidoreductase 2OG-Fe (II) oxygenase* protein from maize adjacent to Sobic.001G166200.

### The Local Linkage Disequilibrium (LD)

LD among the SNPs with significant association to different traits ranged from very low R^2^ for glume cover to high R^2^ for several others with at least one haplotype group in each of the traits (Figure 5). Four haplotype groups in plant height, two in panicle compactness and shape and smut resistance, and a single haplotype block accounted for presence or absence of awns, as well as pericarp color and male sterility were identified.

## Discussion

### Population Stratification and GWAS Model

The population structure generated using hierarchical clustering, Admixture, and principal component analysis identified a clear differentiation across the large Ethiopian landrace collection used for this study (Figure 2, 3). The CMLM (Zhang et al., 2010) that corrects for population structure, marker effect and kinship and reduces confounding effects, was implemented for the association analysis as a more appropriate model. The quantile-quantile (Q-Q) plots (Supplementary Figure S3) validated this assertion with the CMLM showing consistency in reducing -log10(*p*-values) toward the expected level; they controlled false-positives and removed the confounding effects due to population structure.

### Genome-Wide Associations and Identification of Candidate Genes

We conducted GWAS for eight traits related to morphological, agronomic and disease resistance based on cumulative data from different year/location and single year data for fertility reaction in sorghum. Awns, slender bristles located at tip of a glume or lemma in a grass spikelet, are known to be critically important for photosynthesis and transpiration (Tedeschi et al., 2017). The current association study identified a single candidate gene associated to awns on chromosome 3, which has been previously reported for association with grain shape, size and weight in rice (Yan et al., 2013). The result is consistent with earlier classical genetic mapping observations conducted using recombinant inbred lines (RILs), where the gene controlling awn length and presence or absence of awns in sorghum was mapped to 157.9–161.6 cM region also on chromosome 3 (SBI-03) (Hart et al., 2001; Mace and Jordan, 2010). The specific genes that control the occurrence awns have not been determined, although, a number of candidate genes describing awn length and shape have been previously reported in wheat (Yoshioka et al., 2017) as well as barley (Liller et al., 2017).

Panicle compactness and shape is among important descriptors used for panicle morphology in sorghum, as well as in the classification of sorghum to five major races and 10 intermediate sub race groups (Dillon et al., 2007). It is possible that candidate loci identified in this study provide genetic markers to validate the widely-used race taxonomy of sorghum. In general, 15 regions across the sorghum genome were identified for association with panicle compactness and shape. Genomic regions with proteins implicated in cell growth and development; meristem development (Clark et al., 1997) and regulation of floral organ abscission (Jinn et al., 2000), regulation of cell cycle, signal transduction, cell differentiation, and transformation (Liu et al., 2003) were identified. Similarly, genes important for thermo-tolerance (Zhao et al., 2003), plant development and differentiation (Masiero et al., 2002), regulation of abiotic stress tolerance (Perea-Resa et al., 2016), and in electron transport process associated with photosynthesis were identified (Schottler, 2004).

Ethiopian farmers mostly grow sorghums with colored pericarps as they consider them better in nutritional values and are better tolerant to biotic and abiotic stresses. Since local farmers mostly grow local landraces, and maintain mixed seed sources, colored sorghums often tend to prevail in the production landscape of the country. Many of the improved cultivars that came out of sorghum research programs are often white seeded. The loci controlling pericarp color have been well-studied in sorghum and in some cases it was used as a phenotype to validate correct SNP calling, imputation, and GWAS methodology (Brenton et al., 2016). However, previous mapping efforts only determined a rough location of few of the loci over a wider genomic region. Our study provided more specificity by identifying five SNPs within the previously described candidate gene, *YELLOW SEED1* (Y1) on chromosome 1. The Y1 locus harbors two additional MYB genes which share high sequence identity to Y1. We consider this as the first precise mapping of *YELLOW SEED1* locus using natural variants. In addition to Y*ELLOW SEED1* MYB transcription factor that is previously described (Ibraheem et al., 2010; Morris et al., 2013b; Brenton et al., 2016), four additional candidate genes (Sobic.001G398100, Sobic.001G400400, Sobic.001G400300, and Sobic.001G397850) and two other novel loci on chromosome 3 and chromosome 4 were associated with pericarp color. These candidate genes regulate anthocyanin biosynthesis (Selinger and Chandler, 1999; Schwinn et al., 2006; Stommel et al., 2009; Zabala and Vodkin, 2014), stress tolerance (Wang et al., 2015), and cell wall metabolism (Phan et al., 2007). The Sobic.004G280800 gene, similar to anthocyanin biosynthetic gene regulator PAC1, has been described as *Tannin 1*, reported for its role in presence of tannins in sorghum grains (Wu et al., 2012). Sobic.001G398100 encodes a homolog of the maize TRANSPARENT TESTA GLABRA 1 (TTG1), a protein with WD repeat. TTG1 is known to regulate anthocyanin pigmentation in Arabidopsis (Walker et al., 1999) and is a key component of the MYB-bHLH-WDR protein complex which regulates transcription of flavonoid biosynthesis genes (Xu et al., 2015). In addition, Sobic.001G400500 adjacent to Sobic.001G400400, encodes *Dek1*-calpain-like protein, is required for aleurone layer development and anthocyanin accumulation in maize (Becraft and Yi, 2011) suggesting potential role in the control of pericarp color.

In sorghum, panicle exsertion, a portion of the peduncle from the base of the panicle to the ligule of the flag leaf, is an important consideration especially in seed parent line development. A single region on chromosome 6 with a large effect (*R*^2^ = 0.24) on variation in panicle exsertion, with no functional annotation, was identified. Previously, multiple regions on chromosome 1, 2, 3, 6, 7, 9, and 10 were associated to the trait but with smaller effects (0.076 < *R*^2^ < 0.118) (Zhao et al., 2016). Sobic.006G094800 appears to control more than one trait as it is significantly associated with both panicle exsertion and panicle compactness and shape. Interestingly, Sobic.006G095550 was associated with glume cover and panicle compactness and shape, which suggests a potential pleiotropic effect for these two loci, singularly or in tandem, potentially controlling more than one trait. The seemingly correlated nature of these phenotypic traits across sorghum races makes this association, even more plausible.

The Ethiopian sorghum germplasm collection harbors a large diversity for plant height. Overall the germplasm represents dominantly tall, photoperiod sensitive, late flowering, long maturing, and low yielding accessions. The plant height trait is important for sorghum farmers in Ethiopia as the stalk of the crop is utilized for livestock feed, construction material for building fences and as a source of energy for cooking generated by burning the stalk. The genetic basis of plant height in sorghum has been well studied (Yamaguchi et al., 2016). Allelic variation at four loci *dw1*, *dw2*, *dw3* and *dw4* are known to regulate plant height by altering the length of stem internodes (Quinby and Karper, 1954). Recent studies based on classical mapping studies have localized *dw2* locus on chromosome 6 (Mace and Jordan, 2010), whereas sequence-based mapping have identified two QTL, *dw1* and *dw3* on chromosome 9 and chromosome 7, respectively (Mace and Jordan, 2010; Hilley et al., 2016). A GWAS study (Li et al., 2015) using sorghum association panel identified a separate quantitative trait locus (qHT7.1) near the genomic region harboring the known auxin transporter, *dw3* gene in addition to previously known *dw1, dw2, dw3*, and *dw4* genes. Among 12 genomic regions currently identified for association with plant height in our study, only two had closest proximity with previously identified loci. These include QTL at 59.6 Mbp which corresponds to *dw3* (Sobic.007G163800) and another nearby QTL at 56.4 Mb found to correspond with *qHT7.1*, initially mapped at 55.2 Mbp (Li et al., 2015). Association studies using natural variants such as the landrace population used in our study could likely be useful for unraveling the genetic control of plant height and other traits that may have been encumbered through the sorghum conversion program. Our study identified 12 loci associated with plant height on chromosome 2, 5,7,8, and 9. The candidate gene in one of these loci, Sobic.009G052200 on chromosome 9 is about 51 Mbp away from the well characterized (Hilley et al., 2016) *dw1* gene (*Sobic.009G229800*), whereas Sobic.007G161700 on chromosome 7 is 0.20 Mbp away from the previously reported *dw3* (*Sobic.007G163800*) (Mace and Jordan, 2010). Our study implicated multiple candidate genes for plant height on chromosome 7. A MYB domain protein responsible for anthocyanin pigmentation and regulation of cuticle biosynthesis (Schwinn et al., 2006; Zabala and Vodkin, 2014; Bi et al., 2016), and LIPOATE-PROTEIN LIGASE, known to be involved in key metabolic pathways and with abundant expression in leaves and developing seeds (Kang et al., 2007) were identified. Similarly, a CHLOROPHYLL A/B-BINDING protein precursor, important for light harvesting and photo protection (Boldt et al., 2012; Pietrzykowska et al., 2014) was also associated with plant height. Furthermore, an *ETHYLENE-RESPONSIVE TRANSCRIPTION FACTOR (RAP2-7)* reported to suppress the transition to flowering time and confer flowering time delay was identified (Aukerman and Sakai, 2003). The CLV1 receptor kinase-like regulates stem cell proliferation and stem cell maintenance (Nimchuk et al., 2011; Nimchuk, 2017), and TYROSINE/NICOTIANAMINE AMINOTRANSFERASE essential for iron uptake (Takahashi et al., 1999; Beasley et al., 2017) and CYTOCHROME P450 contributing to the biosynthesis of cyanogenic glucoside dhurrin (Bak et al., 2000) were identified. In addition, two candidate genes encoding PfkB type carbohydrate kinase protein family were identified. These two proteins were also reported for their importance in regulation of plant growth and development (Laurie and Halford, 2001; Gilkerson et al., 2012). Most of the candidate genes for plant height that were identified in our study have also been previously reported to have roles in plant growth.

Sorghum smut (*Sporisorium sorghi*) is a major disease in almost all countries where open pollinated cultivars are widely grown, and seed treatment is not affordable. To the best of our knowledge this is the first effort to map sorghum head smut resistance using sequence-based mapping. An earlier mapping study (Mace and Jordan, 2010) aimed at integrating data from previously mapped major genes onto a complete genome map, has mapped a major QTL region for resistance to head smut on chromosome 8. In the current study, seven candidate genes were identified, of which five were annotated with characterized proteins. The candidate genes represented different categories of genes that encode a GLYCOSYLTRANSFERASE 3, important for plant cell wall synthesis and disease resistance (Perrin, 2008; Langlois-Meurinne et al., 2005) and a MYB family transcription factor, essential for anthocyanin pigmentation and regulation of cuticle biosynthesis (Schwinn et al., 2006; Zabala and Vodkin, 2014; Bi et al., 2016). In addition, a MADS box protein which is reported for its involvement in organ development and stress resistance in *Brassica rapa* (Saha et al., 2015) is associated with smut resistance. Interestingly, the AUTOPHAGY-RELATED PROTEIN 8C precursor, required for maintenance of cellular viability under nutrient-limited conditions and for efficient nutrient use in plants (Hanaoka et al., 2002) is consistent with the role of autophagy proteins in plant defense. *RWP-RK* protein with a likely function in disease resistance and reported for its importance in cell differentiation and gametophytic development suggests the role of nutrients in defense (Tedeschi et al., 2017). *MADS-box* gene has been associated to head smut resistance in maize (Wang et al., 2012). Of the all the traits studied, the loci identified for smut explained the lowest percentage of the variation and the environment had a major influence, suggesting the need for additional and more focused studies on this trait on selected lines representing the diverse alleles.

Cytoplasmic genetic male sterility system has widely served as primary mechanism for hybrid in sorghum seed production and is based on A1 (milo) cytoplasm. The current GWAS analysis for fertility reaction is based on a carefully filtered set of landraces that has been unambiguously, phenotyped as either non-restorers or full restorers with landraces scored with partial restoration excluded from the data set. We identified eight SNPs associated with male sterility on chromosome 4 spanning a 44.5 kb genomic region with high LD (Figure 5). The candidate loci were previously reported for their role in stress tolerance (Zentella et al., 1999; Li et al., 2004; Virdi et al., 2015). The association between male sterility and biotic and abiotic factors were reported in earlier studies. For example, strong association was found between male sterility and disease susceptibility in hybrids produced using cytoplasmic-genic male sterility system and attributed to the same gene in maize (Levings, 1990). A significant effect of photoperiod and minimum temperatures on male sterility during the period from panicle initiation to flowering were also reported in hybrid rice (El-Namaky and van Oort, 2017). In addition, the strongest SNP on chromosome 4 (S4_1379552) landed near the transcription factor gene *ABORTED MICROSPORES (AMS)*, underlying male sterility in Maize (Liu et al., 2017), Melon (*Cucumis melo* L.) (Sheng et al., 2017), and Arabidopsis (Sorensen et al., 2003). Cytoplasmic male sterile plants with A1 cytoplasm contain small pointed anthers with normal meiosis, but the AMS gene, a recessive nuclear gene, causes premature tapetal degeneration, reduction in filament elongation and a complete microspore abortion and hence results in male sterility (Sorensen et al., 2003) making a strong case for the candidate gene.

## Conclusion

Our study involved field-based phenotyping and genotyping-by-sequencing of a large and diverse germplasm collection of sorghum landraces from Ethiopia. This approach helped define loci and discover candidate genes underlying the genetic variation in eight important traits. Sorghum germplasm from this region is one of the richest for trait diversity and the genes and alleles underlying those traits. This diversity has evolved under the diverse and unique environmental conditions and also selected by farmers for diverse uses. In addition to helping to better understand the genetic architecture of these traits, the study will enhance sorghum improvement effort around the world, as development of new markers will offer opportunities in improving the efficiency and precision of sorghum breeding. Functional validation of these newly discovered candidate genes is also important to affirm the statistical association results established here based on the GWAS analysis.

## Data Availability

All datasets for this study are included in the manuscript and the Supplementary Files.

## Author Contributions

TeT, GE, and TM conceived and supervised the research work. TM, TeT, GE, GA, FM, KB, AT, HN, and GG initiated and designed the field and genotyping experiments. AS, AN, MM, AG, DL, AB, and HN performed field experiments and prepared leaf tissue samples. GG, HN, AA, and TM analyzed the data. GG wrote manuscript. HN, DL, TaT, TeT, GE, and TM edited the manuscript. All authors have read and approved the final version of the manuscript.

## Conflict of Interest Statement

The authors declare that the research was conducted in the absence of any commercial or financial relationships that could be construed as a potential conflict of interest.

## Footnotes

1 https://purr.purdue.edu/publications/3189/1 (DOI: 10.4231/PYQV-AT79)

2 https://purr.purdue.edu/publications/3189/1 (DOI: 10.4231/PYQV-AT79)

## Supplementary Material

FIGURE S1 Description of the phenotypic traits across1425 sorghum accessions used for the GWAS analysis. **(A–C)** plant height from Bako, Haramaya and combined, respectively, **(D)** presence and absence of awns (1 = awned and 2 = awnless), **(E)** panicle compactness and shape (1 = loose erect, 2 = loose drop, 3 = compact elliptic (erect) and 4 = compact oval or recurved), **(F)** glume covering (1 = grain uncovered, 2 = 25% of grain covered, 3 = 50% of grain covered, 4 = 75% of grain covered, 5 = grain fully covered, 6 = glumes longer than grain), **(G)** pericarp color (1 = white, 2 = yellow, 3 = red, 4 = brown and 5 = buff), **(H)** panicle exsertion score (1 = panicle well exserted with 10 cm between ligule of flag leaf to panicle base, 2 = 2–10 cm exsertion, 3 = less than 2 cm but ligule below the panicle base, 4 = peduncle recurved but panicle is below the ligule and clearly exposed splitting the leaf sheath, 5 = panicle covered by leaf sheath), **(I)** sterility group and **(J)** smut damage score.

FIGURE S2 Manhattan plots across 1425 Ethiopian sorghum landrace collection using 72,190 SNP markers across years/locations for each trait. The blue and red horizontal lines indicate significant false discovery rate (FDR)- adjusted *P* ≤ 0.05 and ≤ 0.01, respectively.

FIGURE S3 Quantile-Quantile (QQ) plots from the GWAS scan **(A)** awns, **(B)** panicle compactness and shape, **(C)** panicle exertion, **(D)** pericarp color, **(E)** glume covering, **(F)** plant height, **(G)** resistance to smut, and **(H)** male sterility.

TABLE S1 Geographic description of the study areas. ^∗^The Köppen-Geiger climate classification; AEZs, agro-ecological zones; m.a.s.l, meter above sea level; Aw, tropical wet and dry or savanna climate; Cwa, humid subtropical climates or mild temperate climates; Cwb, subtropical highland climate.

TABLE S2 Genome-wide association study results describing the most significant SNPs (FDR ≤ 0.05), physical position with corresponding chromosome, MAF, transcript name and biological function across eight traits representing morphological, agronomic, smut resistance, and male sterility traits.
