# Supplementary material for: A Large-Scale Genome-Wide Association Analyses of Ethiopian Sorghum Landrace Collection Reveal Loci Associated With Important Traits
Source: Front Plant Sci. 2019 May 29;10:691. doi: 10.3389/fpls.2019.00691 (PMC6549537; doi:10.3389/fpls.2019.00691)
Supplement: FIGURE S1 — Description of the phenotypic traits across1425 sorghum accessions used for the GWAS analysis. (A–C) plant height from Bako, Haramaya and combined, respectively, (D) presence and absence of awns (1 = awned and 2 = awnless), (E) panicle compactness and shape (1 = loose erect, 2 = loose drop, 3 = compact elliptic (erect) and 4 = compact oval or recurved), (F) glume covering (1 = grain uncovered, 2 = 25% of grain covered, 3 = 50% of grain covered, 4 = 75% of grain covered, 5 = grain fully covered, 6 = glumes longer than grain), (G) pericarp color (1 = white, 2 = yellow, 3 = red, 4 = brown and 5 = buff), (H) panicle exsertion score (1 = panicle well exserted with 10 cm between ligule of flag leaf to panicle base, 2 = 2–10 cm exsertion, 3 = less than 2 cm but ligule below the panicle base, 4 = peduncle recurved but panicle is below the ligule and clearly exposed splitting the leaf sheath, 5 = panicle covered by leaf sheath), (I) sterility group and (J) smut damage score. [file Presentation_1.PPTX]

## Slide 1
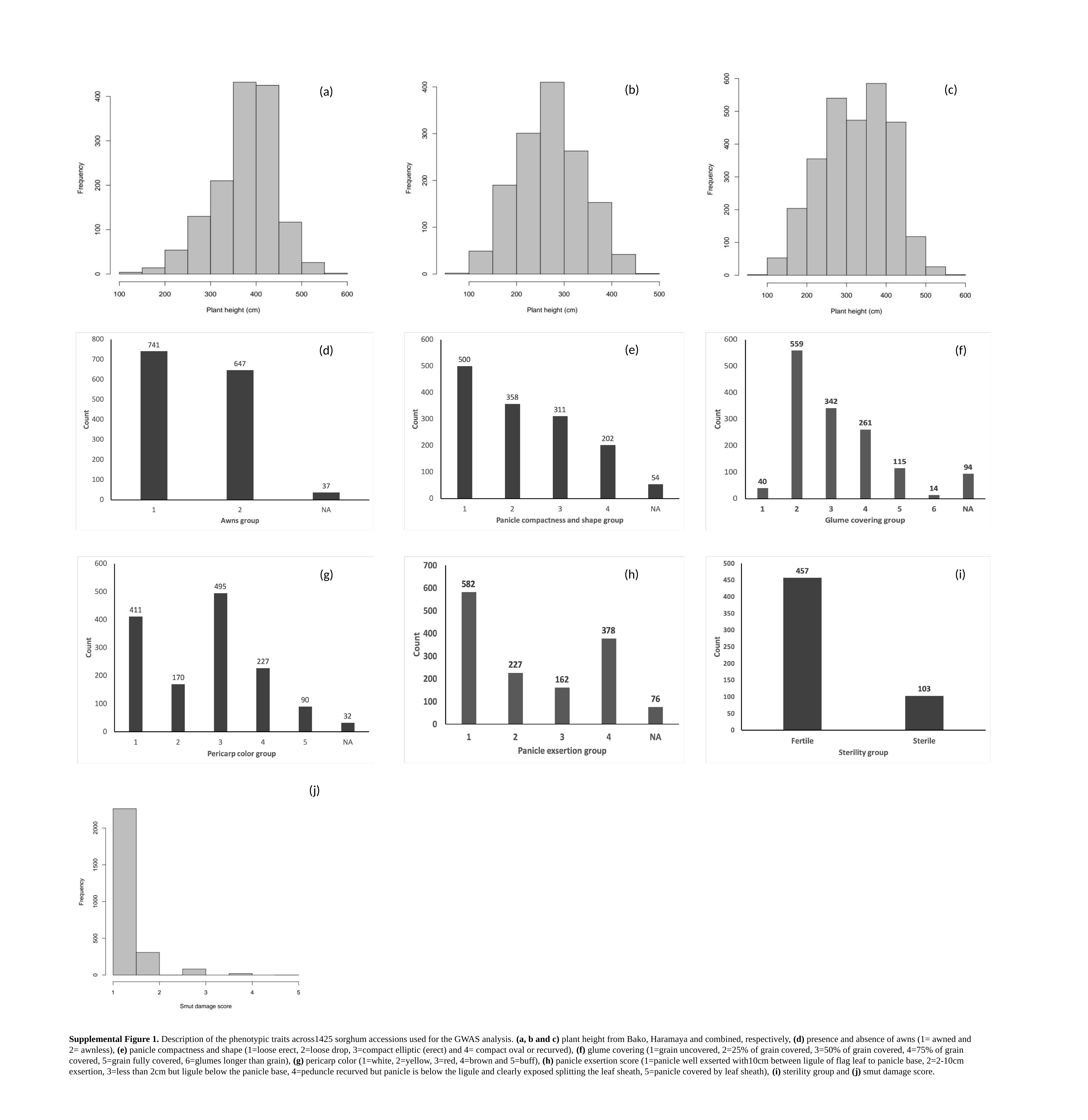

(b)
(c)
(a)
(e)
(d)
(f)
(h)
(i)
(g)
(j)
Supplemental Figure 1. Description of the phenotypic traits across1425 sorghum accessions used for the GWAS analysis. (a, b and c) plant height from Bako, Haramaya and combined, respectively, (d) presence and absence of awns (1= awned and 2= awnless), (e) panicle compactness and shape (1=loose erect, 2=loose drop, 3=compact elliptic (erect) and 4= compact oval or recurved), (f) glume covering (1=grain uncovered, 2=25% of grain covered, 3=50% of grain covered, 4=75% of grain covered, 5=grain fully covered, 6=glumes longer than grain), (g) pericarp color (1=white, 2=yellow, 3=red, 4=brown and 5=buff), (h) panicle exsertion score (1=panicle well exserted with10cm between ligule of flag leaf to panicle base, 2=2-10cm exsertion, 3=less than 2cm but ligule below the panicle base, 4=peduncle recurved but panicle is below the ligule and clearly exposed splitting the leaf sheath, 5=panicle covered by leaf sheath), (i) sterility group and (j) smut damage score.
