# Supplementary material for: A Large-Scale Genome-Wide Association Analyses of Ethiopian Sorghum Landrace Collection Reveal Loci Associated With Important Traits
Source: Front Plant Sci. 2019 May 29;10:691. doi: 10.3389/fpls.2019.00691 (PMC6549537; doi:10.3389/fpls.2019.00691)
Supplement: FIGURE S2 — Manhattan plots across 1425 Ethiopian sorghum landrace collection using 72,190 SNP markers across years/locations for each trait. The blue and red horizontal lines indicate significant false discovery rate (FDR)- adjusted P ≤ 0.05 and ≤ 0.01, respectively. [file Presentation_2.PPTX]

## Slide 1
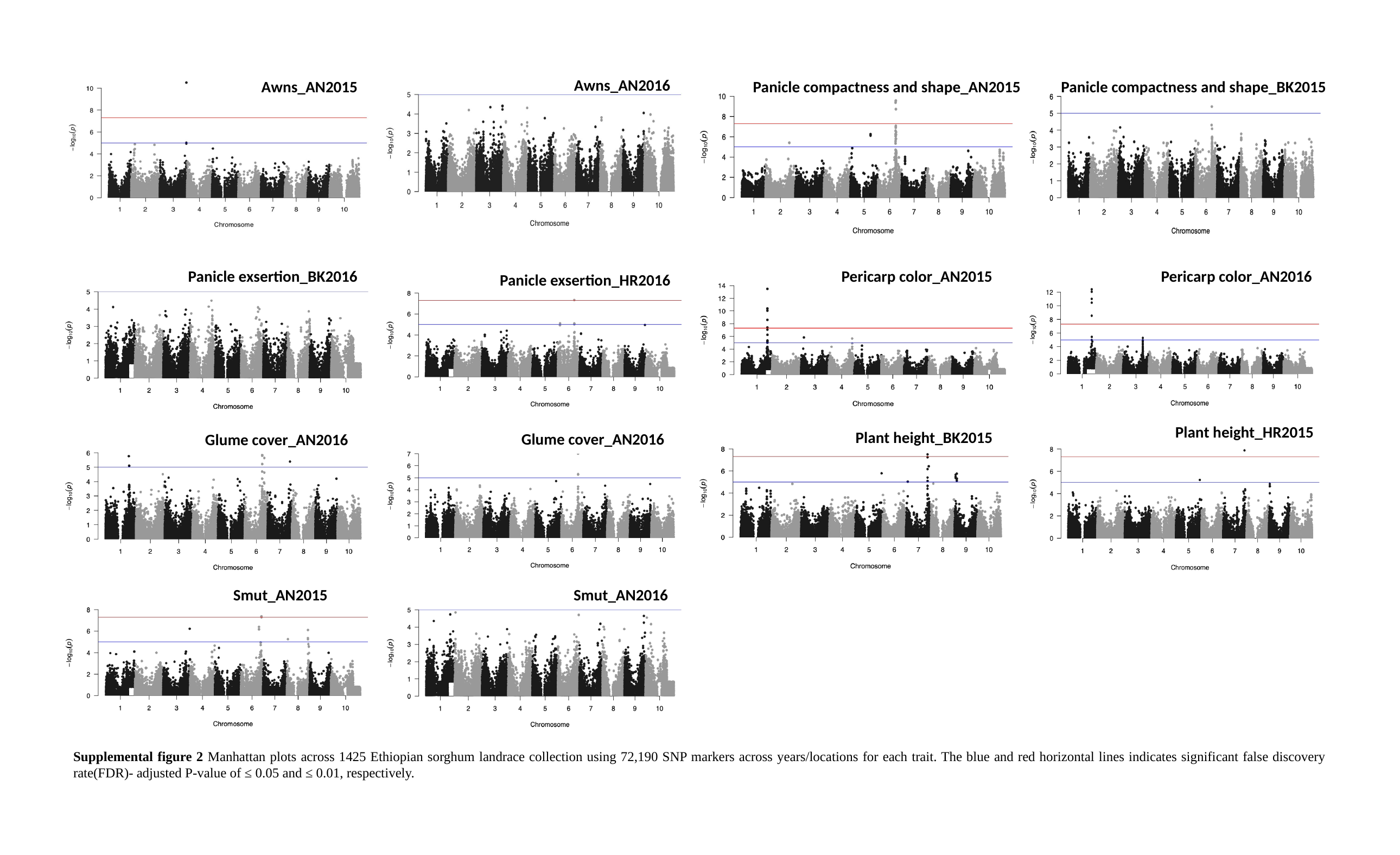

Awns_AN2016
Awns_AN2015
Panicle compactness and shape_BK2015
Panicle compactness and shape_AN2015
Pericarp color_AN2015
Panicle exsertion_BK2016
Panicle exsertion_HR2016
Pericarp color_AN2016
Plant height_HR2015
Plant height_BK2015
Glume cover_AN2016
Glume cover_AN2016
Smut_AN2016
Smut_AN2015
Supplemental figure 2 Manhattan plots across 1425 Ethiopian sorghum landrace collection using 72,190 SNP markers across years/locations for each trait. The blue and red horizontal lines indicates significant false discovery rate(FDR)- adjusted P-value of ≤ 0.05 and ≤ 0.01, respectively.
