# Supplementary material for: A Large-Scale Genome-Wide Association Analyses of Ethiopian Sorghum Landrace Collection Reveal Loci Associated With Important Traits
Source: Front Plant Sci. 2019 May 29;10:691. doi: 10.3389/fpls.2019.00691 (PMC6549537; doi:10.3389/fpls.2019.00691)
Supplement: FIGURE S3 — Quantile-Quantile (QQ) plots from the GWAS scan (A) awns, (B) panicle compactness and shape, (C) panicle exertion, (D) pericarp color, (E) glume covering, (F) plant height, (G) resistance to smut, and (H) male sterility. [file Presentation_3.PPTX]

## Slide 1
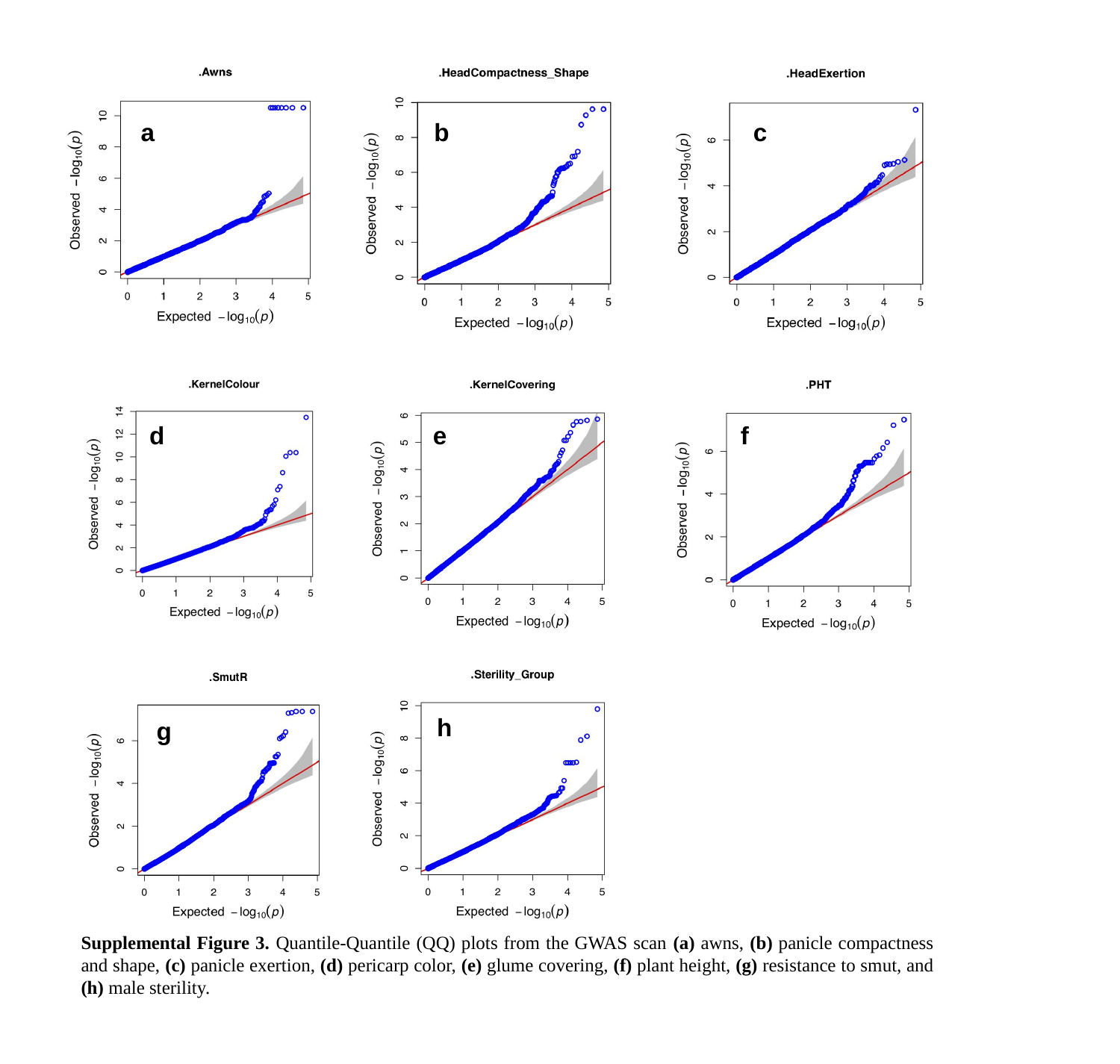

a
b
c
d
e
f
h
g
Supplemental Figure 3. Quantile-Quantile (QQ) plots from the GWAS scan (a) awns, (b) panicle compactness and shape, (c) panicle exertion, (d) pericarp color, (e) glume covering, (f) plant height, (g) resistance to smut, and (h) male sterility.
