# Supplementary material for: A Large-Scale Genome-Wide Association Analyses of Ethiopian Sorghum Landrace Collection Reveal Loci Associated With Important Traits
Source: Front Plant Sci. 2019 May 29;10:691. doi: 10.3389/fpls.2019.00691 (PMC6549537; doi:10.3389/fpls.2019.00691)
Supplement: TABLE S1 — Geographic description of the study areas. ∗The Köppen-Geiger climate classification; AEZs, agro-ecological zones; m.a.s.l, meter above sea level; Aw, tropical wet and dry or savanna climate; Cwa, humid subtropical climates or mild temperate climates; Cwb, subtropical highland climate. [file Table_1.docx]

**Supplemental Table 1** Geographic description of the study areas.

| Geographic description | Bako | Arsi Negelle | Haramaya |
| --- | --- | --- | --- |
| Latitude/longitude | 9°08’N/37°03′E | 7°21’N/38°42′E | 9° 24’N/42°01′E |
| AEZs | Aw* | Cwa* | Cwb* |
| Broader classification of the AEZs | Intermediate  altitude | Intermediate altitude | Highland |
| Altitude (m.a.s.l) | 1642 | 1951 | 2047 |
| Mean annual Temperature | 19.7 °C | 17.7 °C | 17.9 °C |
| Mean annual rainfall | 1281 mm | 915 mm | 799mm |
| Planting Date | 1-15 May | 10- 30 April | 10-30 April |
| Harvesting Date | 15-30 November | 1-15 December | 1-15 December |

*The Köppen-Geiger climate classification; AEZs= Agro-ecological zones; m.a.s.l= meter above sea level; Aw= Tropical wet and dry or [savanna](https://en.wikipedia.org/wiki/Tropical_savanna_climate) climate; Cwa= humid subtropical climates or mild temperate climates; Cwb= Subtropical highland climate
